# Body composition measurement and diabetes – A new perspective on development of antibodies after SARS-CoV-2 booster vaccination

**DOI:** 10.1007/s40200-025-01699-1

**Published:** 2025-10-25

**Authors:** Lukas Van Baal, Susanne Tan, Dagmar Fuhrer, Johanna Reinold, Ulf Dittmer, Melanie Fiedler

**Affiliations:** 1 Department of Endocrinology, Diabetes and Metabolism, University Hospital Essen, University of Duisburg-Essen, Hufelandstr. 55, 45147 Essen, Germany; 2 Department of Nephrology, University Hospital Essen, University of Duisburg- Essen, Essen, Germany; 3 Institute for Virology, University Hospital Essen, University of Duisburg-Essen, Essen, Germany

**Keywords:** Antibody titer, Booster vaccination, COVID-19, Diabetes, Overweight, SARS-CoV-2

## Abstract

**Purpose:**

In patients with SARS-CoV-2 infection overweight and diabetes are associated with poor prognosis. Therefore, effectiveness and tolerance of booster vaccination against SARS-CoV-2 has to be ensured. We examined the impact of overweight and diabetes on anti-SARS-CoV-2-titers and the incidence of side effects after booster vaccination.

**Methods:**

We performed a cross-sectional study with consecutive recruitment and convenience sampling of 100 participants vaccinated three times against SARS-CoV-2. Anti-SARS-CoV-2-titers, immunological parameters and biochemical markers of glucose metabolism were measured. Prevalence of vaccination side effects was assessed by personal interview. Moreover, metabolic status was assessed by performance of body composition measurement and body impedance analysis. The glycemic and metabolic parameters were correlated with the anti-SARS-CoV-2-titers and the incidence of side effects. The results were compared between patient with and without overweight and diabetes.

**Results:**

Levels of anti-SARS-CoV-2-titers did not differ between participants with and without overweight (5756.6 ± 6848.9 vs. 4997.0 ± 5050.3, p.83) or participants with and without diabetes (6681.0 ± 6795.2 vs. 5124.0 ± 5070.4 BAU/ml, *p* = .28). Moreover, the investigated glycemic parameters and parameters of the body composition measurement and body impedance analysis did not present any correlation with the antibody levels. Furthermore, we could reveal that vaccination side effects are more likely to occur in participants without diabetes (OR 2.9 (95%-CI: 0.5–4.3, p.04)).

**Conclusions:**

Overweight and diabetes are not associated with lower anti-SARS-CoV-2-titers after booster vaccination. Parameters of the body composition and body impedance analysis did not reveal any further association regarding development of anti-SARS-CoV-2-titers. Vaccination side effects are more likely to occur in participants without diabetes.

**Supplementary Information:**

The online version contains supplementary material available at 10.1007/s40200-025-01699-1.

## Introduction

In patients with severe acute respiratory syndrome coronavirus type 2 (SARS-CoV-2) infection overweight and diabetes mellitus (DM) are two of the most prominent risk factors associated with a nearly three-fold increased risk for a severe course, need of intensive care unit (ICU) treatment and mortality [1–4]. Therefore, development of a sufficiently high titer of antibodies after the vaccination has to be ensured [5].

As overweight and DM are presenting two conditions of chronic inflammation resulting in a dysregulation of the immune system, impaired vaccine response might be of concern. An impaired vaccine response in patients with overweight and DM has already been demonstrated in case of influenza and pneumococcal vaccination [6, 7]. Studies investigating the vaccine response after vaccination against SARS-CoV-2 in patients with overweight and DM remain inconsistent and are limited regarding the third vaccination against SARS-CoV-2 (= booster vaccination). Sufficiently high as well as impaired antibody levels, which were negatively correlated with the body mass index (BMI) and the level of glycemic control, could be demonstrated [8–11]. However, BMI is not able to distinguish between lean mass and different fat tissues. In consequence, influence of overweight, defined by BMI, on immune response or comorbidities can be over- or underestimated [12].

To evaluate the influence of overweight and DM on the development of antibody titers and on possible vaccination side effects we investigated the vaccine response after booster vaccination against SARS-CoV-2 in a cohort of German healthcare-workers with and without overweight and DM. Moreover, we performed a measurement of the body fat excess by bioelectrical body impedance analysis (BIA) and body composition measurement (BCM) to evaluate the impact of overweight on the antibody development more precisely.

## Materials and methods

### Study design

This single center cross-sectional study was conducted at the University Hospital Essen, which is located in the Rhine-Ruhr metropolitan area, North Rhine Westphalia, with a catchment area of 5 million residents. In 100 participants (50 participants with and 50 participants without DM) vaccinated the third time against SARS-CoV-2 anti-SARS-CoV-2-titers were determined. Moreover, a correlation between glycemic and metabolic parameters and the anti-SARS-CoV-2-titers was analysed. The impact of overweight and DM on the incidence of vaccination side effects was investigated. Regarding the impact of overweight fat tissue composition was measured by BCM and BIA.

## Patient cohort and laboratory tests

We performed a consecutive recruitment and convenience sampling of one hundred participants. All participants were vaccinated against SARS-CoV-2 three times. Participants were recruited by personal address of health staff members and their family members of the Department of Endocrinology, Diabetology and Metabolism of the University Hospital Essen from 01/FEB/2022 to 28/FEB/2022. The participants were aged between 20 and 84 years. The sample size was calculated to ensure a 90.0% confidence interval with a margin of error of 4.9%. Furthermore, we aimed for a balanced study design regarding DM to provide more reliable results. In consequence, of the 100 participants 50 had DM and the other 50 did not. Moreover, we identified 39 participants with normal weight (= BMI < 25.0 kg/m^2^) and 61 with overweight (= BMI *≥* 25.0 kg/m^2^). Furthermore, none of the participants had a prior SARS-CoV-2 infection. Prior SARS-CoV-2 infection was ruled out as follows: by a personal interview with a questionnaire of (1) whether SARS-CoV-2 infection has been present in the past, (2) whether typical symptoms of a SARS-CoV-2 infection have been present in the past and (3) whether a virological test via a mouth or nose swab has been carried out in the past. If a test had been carried out, the participants were also asked to submit the corresponding findings in written form. All participants received either vaccination with Pfizer-BioNTech BNT162b2 or with Moderna mRNA‐1273 in different combinations. After obtaining informed consent, all participants had a blood sample taken and a BIA and BCM carried out once within one day. Afterwards the collected data was analysed. A follow-up examination was not part of the study protocol. Serum samples of the participants were analysed for anti-SARS-CoV-2 IgG against the spike glycoprotein using the approved anti-SARS-CoV-2 IgG CLIA (LIAISON^®^ SARS-CoV-2 TrimericS IgG assay, Diasorin, Saluggia, Italy). The assay has a linear measuring range from 4.81 to 2080 BAU/ml. Values equal or above 33.8 BAU/ml are defined as positive. Samples with anti-SARS-CoV-2-titers above the upper quantification limit were retested in a 1:20 dilution. Furthermore, blood samples for measurement of HbA1c, spontaneous plasma glucose, glomerular filtration rate (GFR), immune profile (flow cytometry) and inflammatory markers were obtained from every patient. All blood samples were immediately transferred to our laboratory department and analysed the same day. Detailed information about the biochemical analyses is provided in the supplemental material.

Individual fat tissue composition was analysed by BIA (Seca body composition analyzer, Seca Hamburg, Germany) and BCM (Lunar iDXA, GE Healthcare, Chicago, USA). Before performance of BIA the participants were asked whether they have a pacemaker or metal implants in their bodies. In these cases we would have refrained from performance of BIA. However, this was not necessary in any of the participants. The participants were scanned once in standing position, with four electrodes at the feet and four electrodes at the hands. The scan lasted 60 s. Total fat mass and skeletal muscle mass were considered as the relevant output of this method and used for intermodal comparison. The BIA device used multi-frequency 8-point stand-on bioelectrical impedance analysis to measure total body water by applying an electrical current of 100 µA to the body. The drop in voltage between sensor electrodes at the hands and feet was used to determine total body water. The software calculates fat mass, fat free mass, skeletal muscle mass and visceral adipose tissue volume from total body water, Weight, height, age and gender. The device measured at 20 frequencies, ranging from 1 to 1000 kHz.

Measurement of body composition was only performed in participants with a BMI *≥* 25.0 kg/m^2^ and in case of female sex pregnancy was excluded prior to BCM. The participants were scanned once in a lying position. The BMC device measured the regional and whole-body bone mineral density, lean and fat tissue mass. Additionally, the following values were derived by calculations: bone mineral content, soft tissue mass, regional soft tissue mass, total soft tissue mass, fat-free mass, regional/total soft tissue mass ratio, percentual fat mass, regional percentual fat mass, total body percentage fat mass, Android percentual fat mass, Gynoid percentual fat mass, Android/Gynoid fat mass ratio (A/G ratio) and BMI with narrow fan-beam densitometer. A typical whole-body scan took approximately 5 to 15 min and exposed the participants to 4.0–5.0 µSv of radiation.

### Statistical analysis

Data were analysed using GraphPad Prism (GraphPad Software Inc., San Diego, CA, USA) and SPSS 27.0 (IBM Corporation, Armonk, NY, USA) software. Results are shown as mean ± standard deviation and range or absolute number and percentage. A value of *p* <.05 was considered statistically significant. Laboratory values below and above the detection limit were set to the lower or higher detection limit, respectively. To be able to examine the influence of DM and overweight on the vaccination response separately we performed two subgroup analyses: (1) Participants with DM (*n* = 50) vs. without DM (*n* = 50) and (2) participants with overweight (*n* = 61) vs. normal weight (*n* = 39). Participants without DM and DM, as well as participants with a BMI < and *≥* 25 kg/m^2^ were compared using chi-squared test for categorical data. Univariate analysis of covariance (ANCOVA) was computed for continuous variables followed by Bonferroni-corrected posthoc tests. Analyses were performed without adjustment as well as adjusted for age, sex, presence of chronic kidney disease (CKD; defined by glomerular filtration rate (GFR) following KDIGO criteria). DM and overweight are often correlated. Therefore, ANCOVA of overweight vs. normal-weight participants was adjusted for a co-existing DM. Vice versa ANCOVA of DM vs. no DM was adjusted for BMI. ANCOVA was performed with age and BMI [for analysis of the DM subcohort] as covariate, and sex, presence of CKD, received vaccine combinations and presence of DM [for analysis of the overweight subcohort] as between-subject factors. Moreover, when analyzing anti-SARS-CoV-2-titers an additional adjustment for the vaccine combinations used (as between-subject factor) was performed. To test whether the mean values of the respective vaccination combinations differ significantly from each other, we performed an analysis of variance (ANOVA). Furthermore, participants were controlled for medication, which could alter development of anti-SARS-CoV-2-titers, e.g. immunosuppressive medication. To assess the association between glycemic status, overweight and vaccination side effects binary logistic regression analysis with calculation of odds ratios (OR) was performed. For calculation the side effect (not occurred = 0 vs. occurred = 1) was used as dependent variable and no DM vs. DM respective normal weight vs. overweight participants as the two independent variables. The analysis was performed unadjusted and adjusted for age, sex, as well as presence of CKD, received vaccine combinations and antihypertensive and lipid lowering medication as covariates, as a change in the occurrence of vaccine side effects is being discussed with regard to the latter two [13, 14]. Furthermore, when analysing participants with DM vs. without DM, analysis was additionally adjusted for BMI and when analysis overweight vs. normal-weight participants analysis was additionally adjusted for DM. Linearity was tested assessed using the Box-Tidwell procedure [13]. Bonferroni-correction was applied to all ten terms in the model [14]. All variables were found to follow a linear relationship. Correlations between predictor variables were low (*r* <.70), indicating that multicolinearity was not a confounding factor in the analysis. Correlation analysis between parameters of glucose metabolism or overweight, respectively, and the anti-SARS-CoV-2-titers at the day of investigation were computed as Pearson’s r.

## Results

### Demographic characteristics

The majority of participants was female (64%). Mean age was 45.5 years (*±* 16.5 years) and mean BMI was 27.8 kg/m^2^ (*±* 6.5 kg/m^2^). The majority (56.0%) of participants was overweight and 26.0% of the participants were obese (data not shown). None of the participants underwent immunosuppressive treatment. As comorbidities hypertension, hypercholesterinaemia and CKD were present (data not shown).

Furthermore, we analysed vaccines schemes in our population: 69% of the participants were vaccinated with the same vaccine three times. A vaccination with a heterologous booster was performed in 26% of the participants. 5% of the participants were vaccinated with different vaccines for the first and second vaccination and vaccination for booster was homologous to the second vaccine. Three times (36.0%) Pfizer-BioNTech BNT162b2 was the most common vaccine combination followed by a vaccination with Moderna mRNA‐1273 three times (33.0%). Overall six different vaccine combinations were present in our study population. For a detailed information about the vaccine combinations see Table 1. Analysis of the anti-SARS-CoV-2-titers revealed no significant difference of titer levels depending on the vaccine combination used (Table 2).

**Table 1:** Overview over vaccine combinations within the study population given as absolute number and percentage

	Booster-vaccination	
First two SARS-CoV-2 vaccinations	Pfizer-BioNTech BNT162b2	Moderna mRNA-1273
Pfizer-BioNTech BNT162b2 - Pfizer‐BioNTech BNT162b2	36 (36.0%)	6 (6.0%)
Moderna mRNA-1273 - Moderna mRNA‐1273	20 (20.0%)	33 (33.0%)
Pfizer-BioNTech BNT162b2 - Moderna mRNA‐1273	0 (0.0%)	1 (1.0%)
Moderna mRNA-1273 - Pfizer‐BioNTech BNT162b2	4 (4.0%)	0 (0.0%)

Results are presented as absolute numbers (percentage affected)

**Table 2:** Anti-SARS-CoV-2-titers (Bau/ml) depending on the vaccine combination used

Vaccine combinations	Anti-SARS-CoV-2-titers (Bau/ml)
Pfizer-BioNTech BNT162b2 Pfizer-BioNTech BNT162b2 Pfizer-BioNTech BNT162b2	4697.3 ± 4363.3
Pfizer-BioNTech BNT162b2 Pfizer-BioNTech BNT162b2 Moderna mRNA-1273	12105.3 ± 11691.2
Pfizer-BioNTech BNT162b2 Moderna mRNA-1273 Moderna mRNA-1273	5520.0 ± 6363.4
Moderna mRNA-1273 Moderna mRNA-1273 Moderna mRNA-1273	6151.1 ± 5921.7
Moderna mRNA-1273 Moderna mRNA-1273 Pfizer-BioNTech BNT162b2	5663.1 ± 5036.6
Moderna mRNA-1273 Pfizer-BioNTech BNT162b2 Pfizer-BioNTech BNT162b2	6677.5 ± 7450.4

Results are presented as mean ± standard deviation. ANOVA revealed no significant difference between the six vaccine combinations used (p.52, F = 0.41)

### Overweight vs. normal-weight: Anti-SARS-CoV-2-titers and immunological parameters

Demographic characteristics did not differ in patients with and without overweight (table S2). BCM participants with overweight presented with significantly increased absolute and relative levels of the overall, the visceral and the subcutaneous fat tissue, as well as higher relative levels of the android and gynoid fat tissue (Table 3). In BIA significantly lower values for resistance and reactance could be revealed in the participants with overweight. Time between booster vaccination and titer analysis did not differ between the two groups, neither did anti-SARS-CoV-2-titers (5756.6 ± 6848.9 vs. 4997.0 ± 5050.3 BAU/ml, p.32; Table 3). Unadjusted analysis of the immunological parameters revealed significant lower absolute levels of lymphocytes, CD3 + T-cells, CD4 + T-cells as well as relative levels of normoblasts. However, these differences were no longer significant after performance of ANCOVA (Table 3).

**Table 3:** Demographic, metabolic and immunological parameters in participants after SARS-CoV-2 booster vaccination in participants with overweight vs normal weight

			Results of ANCOVA or chi square test	
	Overweight (BMI *≥* 25 kg/m^2^) (*N* = 61)	Normal weight (BMI < 25 kg/m^2^) (*N* = 39)	unadjusted	adjusted
BMI (kg/m^2^)	29.2 ± 6.0	25.8 ± 2.6	0.02	0.03
Days since booster vaccination	50.2 ± 21.7	58.6 ± 23.5	0.08	-
Anti-SARS-CoV-2-titers (Bau/ml)	5756.6 ± 6848.9	4997.0 ± 5050.3	0.06	0.32
*Vaccine combinations*				
Pfizer-BioNTech BNT162b2 Pfizer‐BioNTech BNT162b2 Pfizer-BioNTech BNT162b2	23 (38%)	13 (33%)	0.61	-
Pfizer-BioNTech BNT162b2 Pfizer‐BioNTech BNT162b2 Moderna mRNA-1273	5 (8%)	1 (3%)	0.31	-
Pfizer-BioNTech BNT162b2 Moderna mRNA‐1273 Moderna mRNA-1273	0 (0%)	1 (3%)	0.25	-
Moderna mRNA-1273 Moderna mRNA-1273 Moderna mRNA-1273	17 (28%)	16 (41%)	0.18	-
Moderna mRNA-1273 Moderna mRNA-1273 Pfizer-BioNTech BNT162b2	15 (25%)	5 (13%)	0.15	-
Moderna mRNA-1273 Pfizer-BioNTech BNT162b2 Pfizer-BioNTech BNT162b2	2 (3%)	2 (5%)	0.61	-
*Metabolic Parameters*				
Fat tissue (kg)	35.5 ± 12.5	18.2 ± 6.1	< 0.01	< 0.01
Fat tissue (%)	38.1 ± 7.7	28.5 ± 8.0	< 0.01	< 0.01
Non fat tissue (kg)	55.1 ± 11.3	46.1 ± 9.0	< 0.01	< 0.01
Non fat tissue (%)	61.5 ± 7.7	71.5 ± 7.8	< 0.01	< 0.01
Fat tissue index	12.2 ± 4.3	6.7 ± 3.0	< 0.01	< 0.01
Non fat tissue index	18.8 ± 2.2	15.9 ± 2.0	< 0.01	< 0.01
Resistence	581.2 ± 74.3	679.8 ± 77.2	< 0.01	< 0.01
Reactance	52.3 ± 9.5	58.8 ± 7.7	< 0.01	< 0.01
Android fat tissue (%)	44.5 ± 7.9	30.4 ± 10.0	< 0.01	< 0.01
Gynoid fat tissue (%)	39.9 ± 12.6	33.6 ± 7.8	< 0.01	< 0.01
Visceral fat tissue (g)	1418.0 ± 855.7	609.2 ± 596.4	< 0.01	< 0.01
Visceral fat tissue (cm^3^)	1343.0 ± 807.2	535.1 ± 568.5	< 0.01	< 0.01
Subcutaneous fat tissue (g)	2152.0 ± 789.4	1032.0 ± 574.7	< 0.01	0.01
Subcutaneous fat tissue (cm^3^)	1974.0 ± 744.7	948.3 ± 588.3	< 0.01	< 0.01
Subcutaneous fat tissue (cm^2^)	234.1 ± 92.2	115.0 ± 62.3	< 0.01	< 0.01
*Immunological Parameters*				
Normoblasts (%)	0.0 ± 0.0	0.1 ± 0.0	0.04	0.40
Lymphocytes (/nl)	1.9 ± 0.5	2.2 ± 0.7	0.03	0.68
CD3 + T-cells (nl)	1332.5 ± 445.9	1500.2 ± 507.1	0.02	0.98
CD4 + T-cells (/nl)	878.4 ± 92.7	964.7 ± 392.4	0.02	0.51

Results are presented as mean ± standard deviation. For continuous variables p values are given as result of ANCOVA tests on the two subgroups uncorrected and adjusted for age, sex, presence of chronic kidney disease and diabetes

N, total number of available data; BMI, body mass index; kg, kilogram; m^2^, square metre; ml, millilitre; g, gram; cm^3^, cubic centimetres; cm^2^, square centimetres; nl, nanolitres

## D vs. NoD: Anti-SARS-CoV-2-titers and immunological parameters

Participants with DM were significantly older (51.6 *±* 15.0 vs. 39.3 *±* 12.0 years, *p* <.01) and presented a higher BMI (29.2 *±* 5.8 vs. 25.8 *±* 4.9 kg/m^2^, p.02), random plasma glucose (154.1 *±* 64.5 vs. 91.4 *±* 10.9 mg/dl, *p* <.01),HbA1c (7.5 *±* 1.8 vs. 5.2 *±* 3.4%, *p* <.01) and incidence of CKD (26% vs. 8%, p.02). In BCM and BIA no differences within the investigated parameters could be revealed after performance of ANCOVA (Table 4 + table S3). The time between titer analysis and date of vaccination was significantly lower in participants with DM (48.6 ± 23.6 vs. 59.3 ± 22.9 days, p.03). Anti-SARS-CoV-2-titers after booster vaccination did not differ significantly between participants with and without DM (6681.0 ± 6795.2 vs. 5124.0 ± 5070.4 BAU/ml, p.27; Table 4).

**Table 4:** Demographic, metabolic and immunological parameters in participants after SARS-CoV-2 booster vaccination in participants with DM vs. no DM

			Results of ANCOVA or chi square test	
	DM (N = 50)	No DM (N = 50)	unadjusted	Adjusted
Age (years)	51.6 ± 15.0	39.3 ± 12.0	< 0.01	-
BMI (kg/m^2^)	29.2 ± 5.8	25.8 ± 4.9	0.02	0.03
Days since booster vaccination	48.6 ± 23.6	59.3 ± 22.9	0.03	-
Anti-SARS-CoV-2-titer (Bau/ml)	6681.0 ± 6795.2	5124.0 ± 5070.4	0.20	0.27
Plasma-Glucose (mg/dl)	154.1 ± 64.5	91.4 ± 10.9	< 0.01	< 0.001
HbA1c (%)	7.5 ± 1.8	5.2 ± 3.4	< 0.01	< 0.01
HbA1c (mmol/mol)	57.8 ± 18.9	33.8 ± 4.8	< 0.01	< 0.01
Chronic kidney disease	13 (26%)	4 (8%)	0.02	-
*Vaccine combinations*				
Pfizer‐BioNTech BNT162b2 Pfizer‐BioNTech BNT162b2 Pfizer‐BioNTech BNT162b2	17 (34%)	19 (38%)	0.68	-
Pfizer‐BioNTech BNT162b2 Pfizer‐BioNTech BNT162b2 Moderna mRNA‐1273	4 (8%)	2 (4%)	0.40	-
Pfizer‐BioNTech BNT162b2 Moderna mRNA‐1273 Moderna mRNA‐1273	1 (2%)	0 (0%)	0.32	-
Moderna mRNA‐1273 Moderna mRNA‐1273 Moderna mRNA‐1273	13 (26%)	20 (40%)	0.14	-
Moderna mRNA‐1273 Moderna mRNA‐1273 Pfizer‐BioNTech BNT162b2	13 (26%)	7 (14%)	0.14	-
Moderna mRNA‐1273 Pfizer‐BioNTech BNT162b2 Pfizer‐BioNTech BNT162b2	3 (6%)	1 (2%)	0.31	-
*Metabolic Parameters*				
Reactance	50.9 ± 9.3	58.8 ± 7.4	< 0.01	0.06
Phase angle	4.9 ± 0.8	5.2 ± 0.6	0.01	0.28
Phase angle percentile	26.7 ± 31.8	41.8 ± 29.1	0.03	0.33
Visceral fat tissue (g)	1411.0 ± 326.5	582.4 ± 420.7	< 0.01	0.38
Visceral fat tissue (cm^3^)	1303.0 ± 308.5	556.0 ± 123.7	< 0.01	0.54
*Immunological Parameters*				
Leukocytes (/nl)	7.8 ± 3.0	6.8 ± 1.5	0.04	0.13
Neutrophile granulocytes (%)	61.7 ± 9.2	58.3 ± 7.4	0.04	0.36
Lymphocytes (%)	26.9 ± 8.9	30.8 ± 6.8	0.02	0.23
IG (%)	0.4 ± 0.2	0.3 ± 0.1	. < 0.01	0.12
Monocytes(/nl)	0.6 ± 0.2	0.5 ± 0.2	0.01	0.01
CD3 + T-cells (%)	73.5 ± 6.7	76.3 ± 6.8	0.04	0.18
CD4 + T-cells (%)	47.2 ± 8.5	49.7 ± 8.7	0.15	0.01
NK-cells (%)	14.7 ± 8.4	9.8 ± 8.7	0.02	0.04
HLA-DR + T-cells (%)	8.1 ± 2.9	4.2 ± 2.5	0.11	0.04
HLA-DR + T-cells (/nl)	182.4 ± 115.9	111.7 ± 72.6	< 0.01	< 0.01

Results are presented as mean ± standard deviation. For continuous variables p values are given as result of ANCOVA tests on the two subgroups uncorrected and adjusted for age, sex. presence of chronic kidney disease and BMI

DM, diabetes mellitus; No DM, no diabetes mellitus; N, total number of available data; BMI, body mass index; m^2^, square metre; ml, millilitre; mg, milligram; dl, decilitre; g, gram; cm^3^, cubic centimetres; nl, nanolitres; IG; immunoglobulins, NK-cells, natural killer-cells; HLA-DR + cells: human leucocyte antigen DR positive t-cells

Analysis of immune parameters revealed that participants with DM presented significantly higher absolute levels of monocytes, relative levels of natural killer cells as well as absolute levels of HLA-DR + T-cells, however, significant lower relative levels of CD4 + T-cells. Moreover, without adjustment for age, sex and BMI participants with DM presented significantly higher relative levels of leukocytes, neutrophile granulocytes. immunoglobulins and absolute numbers of HLA-DR + T-cells as well as significantly lower relative numbers of CD3 + T-cells.

## Correlations analysis

A weak negative correlation between the anti-SARS-CoV-2-titers and the relative levels of CD3 + T-cells could be demonstrated. Besides, no significant correlations between the anti-SARS-CoV-2-titers after the booster vaccination and investigated immunological or demographical parameters or parameters of the BIA and BCM, respectively, could be demonstrated (table S4).

### Metabolic and glycemic status and association with vaccination side effects

69 participants (69.0%) reported at least one side effect after the booster vaccination. Most common side effects were injection side pain (54.0%), followed by fatigue (37.0%), fever (23.0%) headache (19%), joint pain (19.0%), lymphadenopathy (14.0%) and chills (12.0%). Less frequent reported side effects were dizziness (3.0%), nausea (3.0%) and diarrhoea (1.0%).

**Fig. 1:**
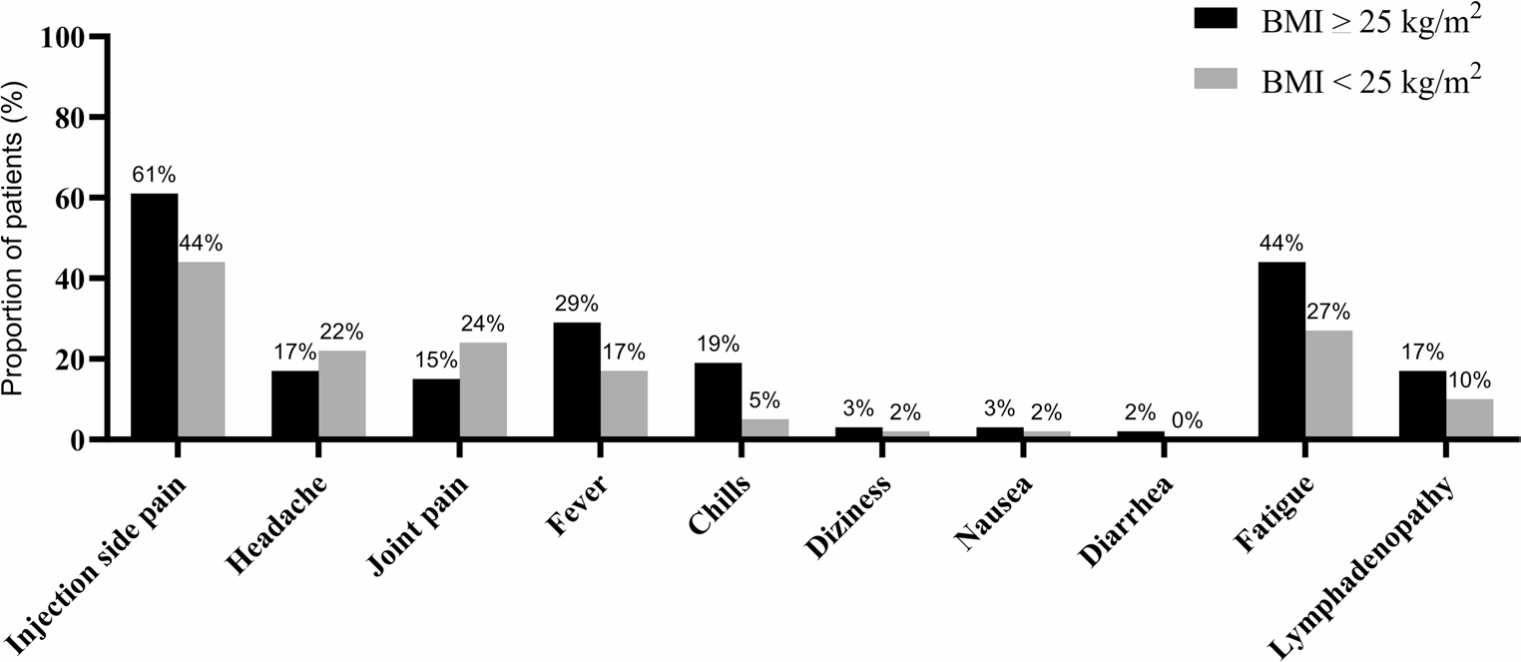
Incidence of side effects after SARS-CoV-2 booster vaccination according to body mass index

Comparison between overweight and normal-weighty participants did not reveal any differences in kind of or numbers of reported side effect (Fig. 1).

**Fig. 2:**
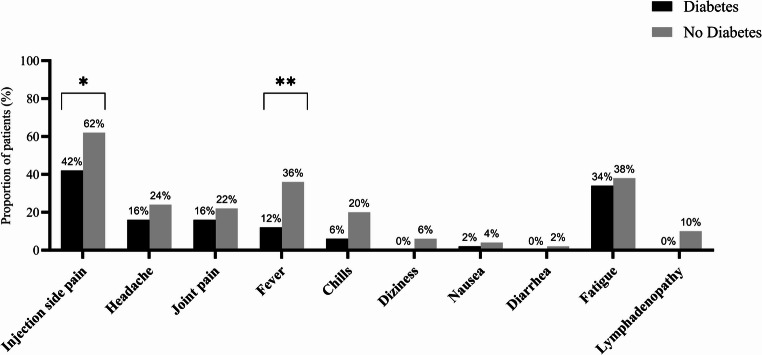
Incidence of side effects after SARS-CoV-2 booster vaccination according to glycemic status. *p<.05, **p<.01

Logistic regression analysis revealed an OR of 2.9 (95%-CI: 0.5–4.3, p.04) for occurrence of at least one side effect in participants without DM compared to participants with DM. In relation to the symptoms reported, there was an OR of 2.7 (95%-CI: 0.2–0.9, p.04; proportion 62.0% vs. 42.0%) for injection side pain and an OR of 3.5 (95%-CI: 0.6–4.7, p.03; proportion 36.0% vs. 12.0%) for fever in participants without DM) (Fig. 2). Regarding participants with overweight vs. normal weight no significant results could be demonstrated.

## Discussion

### Summary

In this cross-sectional study, for the first time the impact of overweight and DM based not only on BMI but also on parameters of BIA and BCM on anti-SARS-CoV-2-titers after booster vaccination was investigated. We could demonstrate that neither overweight nor DM have a negative impact on the anti-SARS-CoV-2-titers.

### Evaluation of anti-SARS-CoV-2-titers after booster vaccination in participants with overweight and DM

We could not demonstrate a significant difference of the anti-SARS-CoV-2-titers between participants with and without overweight after booster vaccination. This result is in line with a large meta-analysis by the Obesity Society investigating the effectiveness of the available vaccines against SARS-CoV- in people with overweight [8]. Furthermore, another meta-analysis reported that all approved SARS-CoV-2 vaccines effectively protect people with overweight in the short term [15]. On the other hand, a review by Hanckova stated that people withoverweight have significantly impaired anti-SARS-CoV-2-titers [9]. Moreover, Takashi et al. revealed a reduced immunogenicity of SARS-CoV-2 vaccine in obese patients with Type 2 DM [16]. Since DM is associated with an over time worsening dysregulation of the immune system [17, 18] the analysis of Takashi et al. might by biased by long time of pre-existing DM in their participants. This might be highlighted by our observation that immunological alterations seen in our participants with and without overweight are no longer significant after adjusting the results for DM. Noteworthy, all former cited studies investigated development of anti-SARS-CoV-2-titers after the first two vaccinations. Studies investigating anti-SARS-CoV-2-titers in overweight patients after booster vaccination are hitherto missing.

Furthermore, studies investigating anti-SARS-CoV-2-titers in overweight people almost exclusively base their definition of overweight on the present BMI. However, immunological alterations in people with overweight are more likely correlated with the present body fat excess than absolute values of BMI [19]. This is also illustrated by our observation of missing influence of the BMI on the immunological parameters studied. Therefore, we investigated the metabolic phenotype of our participants with BIA and BMC. Although, BIA and BCM are much more reliable tools for assessing the body fat excess, only limited data is available about the parameters of BIA and BMC in the context of antibody development after SARS-CoV-2 vaccination [20]. We provide the first study, which investigated possible correlations between anti-SARS-CoV-2-titers and parameters of BIA. Not surprisingly, our participants with overweight presented with nearly threefold increased volumes of the visceral fat tissue. However, we could not reveal any significant correlations between the titer and parameters of the BIA. Noteworthy, the mean BMI in our study was 27.8 kg/m^2^ and only 26 participants fulfilled WHO-criteria of obesity. In consequence, difference in the visceral fat tissue might be not enough pronounced to cause significant immunological dysregulation and in consequence influence the development of antibodies [21, 22]. To the best of our knowledge only one study investigated the correlation between parameters of BCM and anti-SARS-CoV-2-titer after two doses of SARS-CoV-2 mRNA vaccination so far. A negative correlation between anti-SARS-CoV-2-titers and waist circumferences could be revealed, but not concerning the additional parameters of BCM [20]. This is in line with our results.

Regarding DM, not only chronic, but also acute hyperglycaemia at the time of vaccination worsens the immunological response after SARS-CoV-2 vaccination [10, 23, 24]. Furthermore, a recent study of D`Onofrio et al. revealed an impaired anti-SARS-CoV-2-titer in patients with DM even six months after receiving the first two doses of mRNA vaccine, highlighting the relevance of a booster vaccination [25]. We could demonstrate that anti-SARS-CoV-2-titers after mRNA booster-vaccination did not differ between participants with and without DM. Furthermore, our findings suggest that the quality of glycemic control did not influence the anti-SARS-CoV-2-titers. This finding contrasts with Marfella et al. and might be explained due to the absolute and relative higher numbers of patients with DM and an HbA1c > 7.0% in the study of Marfella et al. [23] (298 [63.3%] vs. 26 [52.0%]). In patients with poorly controlled DM the CD4 + cellular response was described to be defective after SARS-CoV-2 vaccination [26, 27]. Although we could demonstrate a significantly impaired relative level of CD4 + T-cells in our participants with DMcompared to without DM (47.2% vs. 49.7%), the resulting clinical relevance of this difference seems unlikely and might explain the non-existent difference in terms of the anti-SARS-CoV-2-titers. Of note, we described a significantly shorter time of 10 days between booster vaccination and analysis of anti-SARS-CoV-2-titers in our participants with DM. However, absolute number of days was 48.6 in participants with DM and 59.3 in participants without DM. In consequence an influence on our titer analysis seems unlikely, since a sufficient development of anti-SARS-CoV-2-titers 28 days after vaccination has been described [28].

### Evaluation of vaccine side effects in participants with DM and overweight

Most frequently reported side effects were injection site pain in nearly half of our participants and fatigue in one third. No serious side effects with the need to consult a physician were reported. These findings are in line with the registration studies of the available vaccines [29, 30].

Regarding overweight patients a study among a Spanish population revealed a significant higher risk of presenting fever, vomiting, diarrhoea and chills in non-overweight patients [31]. In accordance we could demonstrate, that fever, chills and diarrhoea tend to be more prevalent in non-overweight participants. However, multinominal regression analysis did not reveal a significant higher risk for occurrence of side effects in our non-overweight participants. In general, a higher prevalence of side effects after SARS-CoV-2 vaccination in people without overweight might be due to the chronic dysfunctional immune system in overweight people resulting in a lesser pronounced immune response [32, 33]. Probably, the mean BMI of 29.2 kg/m^2^ and body fat excess of our participants with overweight might be not high enough to cause clinically relevant immunological alterations. In our analysis, we did not find significantly differing immunologic parameters between people with and without overweight.

The only study investigating the side effects after SARS-CoV-2 vaccination in patients with DM demonstrated a significant higher prevalence only for injection site pain in patients with DM [34]. In contrast, we could demonstrate that the reported side effects tending to be less prevalent in participants with DM. The inconsistent results compared to Riad et al. might be explained by the significant different population sizes and the missing performance of an ANCOVA in the study of Riad et al., as female sex and lower age are associated with a higher rate of side effects [35, 36]. Moreover, odds ratio for the occurrence of at least one side effect in participants without DM was 1.4 times higher than in participants with DM. A cause might be the impaired and dysfunctional immunological biomarkers in patients with DM, leading to a lesser pronounced immune response after vaccination [26, 27]. Accordingly, our patients with DM presented with a significantly lower incidence of fever as objective symptom of systemic immune reaction and with a significantly lower incidence of injection side pain as possible symptom of a local inflammation. Furthermore, our participants with DM presented higher levels of HLA-DR + T-cells and monocytes as well as relative levels of natural killer cells, which are associated with immune dysregulation during an acute SARS-CoV-2 infection [20, 37]. However, the difference in monocyte levels (0.5/nl vs. 0.6/nl) seems unlikely to cause a considerable clinical alteration. Overall the slight disturbance of some immunological parameters in people with DM in our study had no impact on the effectiveness of the booster vaccination.

### Strengths and limitations

To the best of our knowledge, this is the first independent study dealing with the development of anti-SARS-CoV-2-titers and vaccine side effects after the SARS-CoV-2 mRNA booster vaccination in Germany. Furthermore, we offer a detailed insight of the participant’s individual metabolic phenotype by performance of BMC and BIA. In consequence, we could correlate a huge number of metabolic parameters with the anti-SARS-CoV-2-titers, resulting in an improved internal validity and enhancing precision of investigating the concrete influence of an impaired metabolic status.

The external validity of this study is limited because of the small population size (*n* = 100) and the unequally distribution across gender. Furthermore, six different vaccine combinations were used in our study population. No significant influence of the vaccine combinations on the anti-SARS-CoV-2-titers could be revealed in the ANCOVA or for the occurrence of side effects in the logistic regression analysis. The two different vaccines differ in dosage. However, a recent systematic review did not reveal any significant difference in vaccine efficiency and safety comparing Pfizer-BioNTech BNT162b2 and Moderna mRNA‐1273 [38]. Moreover, the side effects except fever were reported subjectively and were influenced by individual perception of pain and fatigue. These confounders are difficult to standardize and may impact reported side effects.

## Conclusion

Presence of overweight, increased visceral fat tissue and DM are not associated with lower anti-SARS-CoV-2-titers after mRNA SARS-CoV-2 booster vaccination. Moreover, side effects after the booster vaccination tend to be less prevalent in people with overweight and DM.

Results are presented as mean ± standard deviation. For continuous variables p values are given as result of ANCOVA tests on the two subgroups uncorrected and adjusted for age, sex, presence of chronic kidney disease and BMI.

DM, diabetes mellitus; No DM, no diabetes mellitus; N, total number of available data; BMI, body mass index; m^2^, square metre; ml, millilitre; mg, milligram; dl, decilitre; g, gram; cm^3^, cubic centimetres; nl, nanolitres; IG; immunoglobulins, NK-cells, natural killer-cells; HLA-DR + cells: human leucocyte antigen DR positive t-cells.

## Supplementary Information

Below is the link to the electronic supplementary material.

Supplementary Material 1

## Data Availability

No datasets were generated or analysed during the current study.
