# Supplementary material for: Body composition measurement and diabetes – A new perspective on development of antibodies after SARS-CoV-2 booster vaccination
Source: J Diabetes Metab Disord. 2025 Oct 25;24(2):250. doi: 10.1007/s40200-025-01699-1 (PMC12553712; doi:10.1007/s40200-025-01699-1)
Supplement: Supplementary file 1 — Supplementary Material 1 [file 40200_2025_1699_MOESM1_ESM.docx]

*Supplemental table 1: Baseline characteristics of the whole study population*

|  | Total |
| --- | --- |
|  |  |
| Age (years) | 45.5 ± 16.5 |
| Female | 64 (64%) |
| BMI (kg/m^2^) | 27.8 ± 6.5 |
| Days since booster vaccination | 45.0 ± 16.5 |
| Anti-SARS-CoV-2-titers (BAU/ml) | 5902.2 ± 6015.9 |
| Plasma-Glucose (mg/dl) | 122.8 ± 55.8 |
| HbA1c (%) | 6.3 ± 1.7 |
| HbA1c (mmol/mol) | 45.8 ± 18.3 |
| Chronic kidney disease | 17 (17%) |
| Antihypertensive medication | 23 (23%) |
| Statin treatment | 13 (13%) |
| *Metabolic Parameters* |  |
| Fat tissue (kg) | 28.1 ± 12.8 |
| Fat tissue (%) | 34.1 ± 9.3 |
| Non fat tissue (kg) | 51.1 ± 11.2 |
| Non fat tissue (%) | 65.7 ± 9.0 |
| Fat tissue index | 9.8 ± 4.6 |
| Non fat tissue index | 17.5 ± 2.5 |
| Mass of skeletal muscle (kg) | 26.3 ± 23.6 |
| Resistence | 621.2 ± 89.8 |
| Reactance | 54.9 ± 9.2 |
| Phase angle | 5.1 ± 0.7 |
| Phase angle percentile | 34.3 ± 31.2 |
| ECW/TBW | 44.2 ± 3.8 |
| Android fat tissue (%) | 38.3 ± 10.9 |
| Gynoid fat tissue (%) | 37.2 ± 10.2 |
| A/G Ratio | 26.4 ± 139.2 |
| Visceral fat tissue (l) | 2.1 ± 3.5 |
| Visceral fat tissue (g) | 1063.0 ± 845.7 |
| Visceral fat tissue (cm^3^) | 989.0 ± 806.2 |
| Visceral fat tissue (cm^2^) | 163.1 ± 272.8 |
| Subcutaneous fat tissue (g) | 1618.1 ± 785.1 |
| Subcutaneous fat tissue (cm^3^) | 1487.3 ± 779.3 |
| Subcutaneous fat tissue (cm^2^) | 177.5 ± 87.8 |
| *Immunological Parameters* |  |
| Leukocytes (/nl) | 7.3 ± 2.4 |
| Neutrophile granulocytes (%) | 60.0 ± 8.4 |
| Lymphocytes (%) | 28.9 ± 8.1 |
| Monocytes (%) | 8.0 ± 2.2 |
| Eosinophiles (%) | 1.9 ± 1.3 |
| Basophiles (%) | 0.7 ± 0.3 |
| IG (%) | 0.3 ± 0.2 |
| Normoblasts (%) | 0.0 ± 0.0 |
| Neutrophile granulocytes (/nl) | 4.4 ± 2.0 |
| Lymphocytes (/nl) | 2.0 ± 0.7 |
| Monocytes(/nl) | 5.6 ± 1.9 |
| Eosinophiles (/nl) | 1.4 ± 1.0 |
| Basophiles (/nl) | 0.4 ± 0.3 |
| IG (/nl) | 0.4 ± 0.1 |
| Normoblasts (/nl) | 0.0 ± 0.0 |
| CD3+ T-cells (%) | 74.9 ± 6.8 |
| CD3+ T-cells (nl) | 1435.0 ± 491.5 |
| CD19+ T-cells (%) | 33.7 ± 30.4 |
| CD19+ T-cells (/nl) | 249.0 ± 132.7 |
| CD4+ T-cells (%) | 48.5 ± 8.6 |
| CD4+ T-cells (/nl) | 928.4 ± 363.1 |
| CD8+ T-cells (%) | 25.2 ± 12.5 |
| CD8+ T-cells (/nl) | 498.7 ± 505.9 |
| CD4/CD8 | 2.2 ± 1.2 |
| NK-cells (%) | 12.2 ± 9.6 |
| NK-cells (/nl) | 228.7 ± 139.1 |
| Cytotoxic T-cells (%) | 3.5 ± 2.1 |
| HLA-DR+ T-cells (%) | 4.6 ± 2.8 |
| HLA-DR+ T-cells (/nl) | 147.0 ± 102.5 |

Results are presented as mean±standard deviation or total number (percentage affected); BMI, body mass index; kg, kilogram; m^2^, square metre; ml, millilitre; mg, milligram; dl, decilitre; kg, kilogram; l, litres; g, gram; cm^3^, cubic centimetres; cm^2^, square centimetres; nl, nanolitre; IG, immunoglobulins

*Supplemental table 2: Non-significant differing demographic and immunological related characteristics in participants after SARS-CoV-2 booster vaccination in participants with overweight vs. normal weight*

|  |  |  | Results of ANOVA or chi square test | |
| --- | --- | --- | --- | --- |
|  | Overweight (BMI > 25 kg/m^2^)  (N=61) | Normal weight (BMI < 25 kg/m^2^)  (N=39) | unadjusted | adjusted |
| Age (years) | 51.0±16.8 | 43.9±16.8 | .07 | - |
| Female | 21 (54%) | 40 (67%) | .41 | - |
| Days since booster vaccination | 50.2±21.7 | 58.6±23.5 | .08 | - |
| Anti-SARS-CoV-2-titers (BAU/ml) | 5756.6±6848.9 | 4997.0±5050.3 | .06 | .83 |
| Plasma-Glucose (mg/dl) | 134.9±55.8 | 117.6±54.4 | .50 | - |
| HbA1c (%) | 6.7±1.9 | 6.1±1.3 | .33 | .97 |
| HbA1c (mmol/mol) | 50.6±20.1 | 43.3±16.3 | .28 | .24 |
| Chronic kidney disease | 10 (16%) | 7 (18%) | .80 | - |
| Antihypertensive medication | 17 (28%) | 6 (15%) | .15 | - |
| Statin treatment | 10 (16%) | 3 (8%) | .25 | - |
| *Metabolic Parameters* |  |  |  |  |
| Mass of skeletal muscle (kg) | 26.2±6.5 | 27.0±29.5 | .89 | .82 |
| Phase angle | 5.2±0.8 | 5.0±0.6 | .23 | .06 |
| Phase angle percentile | 36.7±32.8 | 30.6±29.7 | .39 | .09 |
| ECW/TBW | 44.5±5.1 | 43.5±4.0 | .22 | .17 |
| A/G Ratio | 1.7±0.3 | 4.1±1.7 | .55 | .53 |
| Visceral fat tissue (l) | 2.8±4.3 | 1.0±0.8 | .10 | .57 |
| Visceral fat tissue (cm^2^) | 222.7±88.6 | 94.8±32.6 | .12 | .19 |
| *Immunological Parameters* |  |  |  |  |
| Leukocytes (/nl) | 7.8±2.8 | 7.2±2.2 | .58 | .58 |
| Neutrophile granulocytes (%) | 61.8±8.2 | 58.7±84.8 | .20 | .83 |
| Lymphocytes (%) | 26.7±7.9 | 30.5±8.0 | .08 | .58 |
| Monocytes (%) | 8.4±2.7 | 7.8±1.8 | .34 | .52 |
| Eosinophiles (%) | 2.0±1.5 | 1.9±1.1 | .94 | .39 |
| Basophiles (%) | 0.6±0.3 | 0.8±0.4 | .18 | .83 |
| IG (%) | 0.4±0.2 | 0.3±0.2 | .05 | .06 |
| Neutrophile granulocytes (/nl) | 4.6±1.8 | 4.2±1.7 | .31 | .80 |
| Monocytes(/nl) | 0.6±0.2 | 0.6±0.3 | .69 | .89 |
| Eosinophiles (/nl) | 0.1±0.0 | 0.1±0.0 | .67 | .65 |
| Basophiles (/nl) | 0.0±0.0 | 0.0±0.0 | .18 | .82 |
| IG (/nl) | 0.0±0.0 | 0.1±0.0 | .43 | .49 |
| Normoblasts (/nl) | 0.0±0.0 | 0.0±0.0 | .14 | .39 |
| CD3+ T-cells (%) | 73.7±7.1 | 75.7±65.9 | .10 | .52 |
| CD19+ T-cells (%) | 12.6±7.7 | 11.0±3.3 | .18 | .72 |
| CD19+ T-cells (/nl) | 278.5±112.9 | 248.6±126.6 | .65 | .10 |
| CD4+ T-cells (%) | 52.0±14.3 | 48.2±28.7 | .47 | .12 |
| CD8+ T-cells (%) | 23.5±14.1 | 25.4±11.3 | .99 | .20 |
| CD8+ T-cells (/nl) | 382.2±113.6 | 476.0±186.6 | .95 | .08 |
| CD4/CD8 | 2.2±1.1 | 2.3±1.5 | .23 | .39 |
| NK-cells (%) | 13.2±11.4 | 11.8±6.8 | .25 | .82 |
| NK-cells (/nl) | 243.3±28.9 | 254.8±115.0 | .73 | .85 |
| Cytotoxic T-cells (%) | 1.9±0.9 | 2.1±1.1 | .80 | .30 |
| HLA-DR+ T-cells (%) | 7.2±3.1 | 8.6±2.4 | .33 | .45 |
| HLA-DR+ T-cells (/nl) | 144.7±62.3 | 121.9±79.5 | .53 | .67 |

Results are presented as mean±standard deviation or total number (percentage affected); BMI, body mass index; kg, kilogram; m^2^, square metre; N, total number of available data; ml, millilitre; mg, milligram; dl, decilitre; l, litres; cm^2^, square centimetres; nl, nanolitre; IG, immunoglobulins; NK-cells, natural killer-cells; HLA-DR+ cells: human leucocyte antigen DR positive t-cells

*Supplemental table 3: Non-significant differing demographic, metabolic and immunological related characteristics in participants after SARS-CoV-2 booster vaccination in participants with DM vs. no DM*

|  |  |  | Results of ANOVA or chi square test | |
| --- | --- | --- | --- | --- |
|  | DM  (N=50) | No DM  (N=50) | unadjusted | adjusted |
| Female | 29 (58%) | 31 (62%) | .41 | **-** |
| Antihypertensive medication | 14 (28%) | 9 (18%) | .24 | - |
| Statin treatment | 9 (18%) | 4 (8%) | .14 | - |
| *Metabolic Parameters* |  |  |  |  |
| Fat tissue (kg) | 30.5±14.2 | 25.7±11.0 | .09 | .07 |
| Fat tissue (%) | 35.6±9.1 | 32.7±9.3 | .16 | .19 |
| Non fat tissue (kg) | 52.7±11.9 | 49.6±10.5 | .21 | .70 |
| Non fat tissue (%) | 64.5±9.1 | 66.8±8.9 | .23 | .07 |
| Fat tissue index | 10.8±5.1 | 8.9±3.9 | .06 | .54 |
| Non fat tissue index | 17.9±2.7 | 17.1±2.4 | .16 | .14 |
| Mass of skeletal muscle (kg) | 29.5±32.9 | 23.2±6.1 | .23 | .10 |
| Resistence | 599.9±83.0 | 642.0±92.3 | .30 | .20 |
| ECW/TBW | 44.7±4.7 | 43.6±2.6 | .20 | .32 |
| Android fat tissue (%) | 40.1±1.1 | 35.6±6.2 | .12 | .87 |
| Gynoid fat tissue (%) | 36.3±6.0 | 38.4±7.3 | .45 | .44 |
| A/G Ratio | 3.1±2.5 | 2.1±4.6 | .82 | .75 |
| Visceral fat tissue (l) | 2.1 ± 1.5 | 2.1 ± 4.6 | .48 | .33 |
| Visceral fat tissue (cm^2^) | 171.3±53.5 | 151.8±393.8 | .81 | .50 |
| Subcutaneous fat tissue (g) | 1729.0±150.0 | 1472.0±641.8 | .25 | .71 |
| Subcutaneous fat tissue (cm^3^) | 1612.0±145.0 | 1323.0±651.6 | .19 | .69 |
| Subcutaneous fat tissue (cm^2^) | 191.8±90.0 | 158.4±70.4 | .19 | .49 |
| ***Immunological Parameters*** |  |  |  |  |
| Monocytes (%) | 8.2±2.7 | 7.8±1.6 | .34 | .68 |
| Eosinophiles (%) | 2.3±1.3 | 1.8±1.3 | .38 | .55 |
| Basophiles (%) | 0.7±0.4 | 0.7±0.3 | .99 | .55 |
| Normoblasts (%) | 0.0±0.0 | 0.0±0.0 | .16 | .25 |
| Neutrophile granulocytes (/nl) | 4.8±2.5 | 4.0±1.2 | .05 | .09 |
| Lymphocytes (/nl) | 2.0±0.8 | 2.0±0.6 | .89 | .34 |
| Eosinophiles (/nl) | 0.2±0.1 | 0.1±0.1 | .11 | .12 |
| Basophiles (/nl) | 0.1±0.0 | 0.1±0.0 | .34 | .51 |
| IG (/nl) | 0.1±0.0 | 0.0±0.0 | .11 | .45 |
| Normoblasts (/nl) | 0.0±0.0 | 0.0±0.0 | .23 | .36 |
| CD3+ T-cells (nl) | 1409.0±553.4 | 1461.0±424.8 | .60 | .64 |
| CD19+ T-cells (%) | 13.3±9.2 | 15.4±11.9 | .29 | .67 |
| CD19+ T-cells (/nl) | 259.7±249.1 | 238.2±114.5 | .42 | .16 |
| CD4+ T-cells (/nl) | 909.3±393.7 | 947.4±332.7 | .60 | .94 |
| CD8+ T-cells (%) | 25.1±12.5 | 25.4±12.6 | .92 | .90 |
| CD8+ T-cells (/nl) | 546.5±693.0 | 450.9±179.7 | .35 | .21 |
| CD4/CD8 | 2.3±1.3 | 2.2±0.8 | .67 | .38 |
| NK-cells (/nl) | 253.0±178.1 | 204.5±80.3 | .08 | .15 |
| Cytotoxic T-cells (%) | 3.4±2.1 | 3.6±2.0 | .65 | .69 |

Results are presented as mean±standard deviation or total number (percentage affected); D, diabetes; NoD, no diabetes; N, total number of available data; kg, kilogram; ECW, extracellular water; TBW, total body water; A/G ratio, ratio between relative android and relative gynoid fat tissue; l, litres; cm^2^, square centimetres; g, gram; cm^3^, cubic centimeters; nl, nanolitre; IG, immunoglobulins; NK-cells, natural killer-cells

*Supplemental table 4: Correlation analysis of metabolic and immunologic variables and* anti-SARS-CoV-2-titers

| Variable | Spearmans rho | p-value |
| --- | --- | --- |
| Age (years)  BMI (kg/m2)  Fat tissue (kg)  Fat tissue (%)  Non fat tissue (kg)  Non fat tissue (%)  Fat tissue index  Non fat tissue index  Mass of skeletal muscle (kg)  Resistence  Reactance  Phase angle  Phase angle percentile  ECT/TBW  Android fat tissue (%)  Gynoid fat tissue (%)  A/G Ratio  Visceral fat tissue (g)  Visceral fat tissue (cm3)  Visceral fat tissue (cm2)  Subcutaneous fat tissue (g)  Subcutaneous fat tissue (cm3)  Subcutaneous fat tissue (cm2)  Days since booster vaccination  Leukocytes (/nl)  Neutrophile granulocytes (%)  Lymphocytes (%)  Monocytes (%)  Eosinophiles (%)  Basophiles (%)  IG (%)  Normoblasts (%)  Neutrophile granulocytes (/nl)  Lymphocytes (/nl)  Monocytes(/nl)  Eosinophiles (/nl)  Basophiles (/nl)  IG (/nl)  Normoblasts (/nl)  CD3+ T-cells (%)  CD3+ T-cells (nl)  CD19+ T-cells (%)  CD19+ T-cells (/nl)  CD4+ T-cells (%)  CD4+ T-cells (/nl)  CD8+ T-cells (%)  CD8+ T-cells (/nl)  CD4/CD8  NK-cells (%)  NK-cells (/nl)  Cytotoxic T-cells (%)  HLA-DR+ T-cells (%)  HLA-DR+ T-cells (/nl)  Plasma-Glucose (mg/dl)  HbA1c (%)  HbA1c (mmol/mol) | 0.03  0.19  0.21  0.16  0.10  -0.15  0.17  0.12  0.00  -0.10  -0.06  0.01  0.02  0.06  0.03  0.10  0.06  -0.10  -0.12  -0.06  -0.11  -0.09  -0.05  -0.01  -0.04  -0.01  0.04  -0.02  -0.13  -0.06  0.00  -0.09  0.04  0.02  -0.04  -0.01  -0.06  -0.03  -0.06  -0.23  -0.09  0.11  0.12  0.06  -0.05  -0.12  -0.04  -0.13  0.09  0.14  0.03  -0.09  -0.09  0.08  0.02  0.03 | .79  .08  .05  .15  .37  .18  .12  .26  .98  .37  .58  .91  .86  .54  .60  .55  .82  .45  .66  .51  .41  .68  .46  .51  .75  .96  .68  .89  .66  .82  .20  .53  .98  .38  .67  .87  .68  .54  .58  .02  .53  .37  .24  .62  .79  .23  .65  .25  .66  .17  .77  .38  .40  .41  .83  .79 |

Results of linear regression are presented as Spearman Rho correlation coefficient with Rho < .3 small or negligible, ~.5 moderate and > .7 large strength of association and p-values * p<.05. BMI, body mass index; kg, kilogram; m^2^, square meters; ECT/TBW, ratio between extracellular water and total body water; A/G ratio, ratio between relative android and relative gynoid fat tissue; g, gram; cm^3^, cubic centimetres, cm^2^, square centimetres; nl, nalolitre; IG, immunoglobulins; NK-cells, natural killer-cells; HLA-DR+ cells: human leucocyte antigen DR positive t-cells; HbA1c, hemoglobin A1c

Supplemental methods

Biochemical analysis

HbA1c was determined in EDTA blood samples with the Tosoh Automated Glycohemoglobin Analyzer HLC-723G8 (Tosoh Bioscience, Tokyo, Japan) by high performance liquid chromatography (HPLC) using a three-step salt gradient with a measuring range from 3 mmol/mol (2.4%) to 220 mmol/mol (22.3%).

Glucose was determined in NaF plasma blood samples with the Atellica CH GluH_3 (Siemens Healthcare Diagnostics Inc., Germany) by photometry. Glucose is phosphorylated with ATP by hexokinase. Then the glucose-6-phophate is oxidated by a glucose-6-phopshate dehydrogenase with reduction of NAD to NADH. Photometry is performed by 340 nm with deduction of the extinction of the buff.

The glomerular filtration rate (GFR) was determinated as GFR (ml/min/1,73m^2^) = 186 x (([creatinine(mg/dl)])-1,154 x (age(years))-0,203 x (0,742 in case of female participants). Creatinine was measured in serum blood samples with the Atellica CH (Siemens Healthcare Diagnostics Inc., Germany). Creatinine reacts with picric acid and forms a red colour complex. The rate of complex formation is determined photometrically at 505/ 571 nm.

Leukocytes were counted in EDTA blood samples with the XN-1000 (Sysmex Cop., Japan) in a WNR-canal. Leukocytes are presented by fluorescence intensity and cell volume in a scattergram and are summed up.

The cellular immune status was determined in EDTA blood samples with the FC 500 flow cytometry system (Beckman Coulter GmbH, Germany) using tetra panel multicolor mixtures of anti-CD3/CD4/CD8/CD45 antibodies, FITC/PE conjugated (Beckman Coulter Cat# 6607013, RRID: AB_1575971; https://antibodyregistry.org/search?q=AB_157597) and anti-CD3/CD19/CD45/CD56 antibodies, FITC/PE conjugated (Beckman Coulter Cat# 6607073, RRID: AB_1575973; https://antibodyregistry.org/search?q=AB_1575973). SIL-2 was determined in serum blood samples with the Immulite 2000 XPi (Siemens Healthcare Diagnostics Inc., Germany) by a sequential solid phase chemiluminescence immunoassay (Siemens Cat# LKIP1-319, RRID: AB_2904509; https://antibodyregistry.org/search?q=AB_2904509).
